# Program evaluation of a student-led peer support service at a Canadian university

**DOI:** 10.1186/s13033-021-00479-7

**Published:** 2021-05-31

**Authors:** Rahul Suresh, Zoe Karkossa, Jérémie Richard, Maharshee Karia

**Affiliations:** 1 Montreal Neurological Institute, McGill University, Montreal, Canada; 2 Department of Neurology and Neurosurgery, McGill University, Montreal, Canada; 3 Department of Educational and Counselling Psychology, McGill University, Montreal, Canada; 4 McGill University, Montreal, Canada

**Keywords:** Peer support, University, College, Mental health, Stress, Academic institutions, Support, Students

## Abstract

**Background:**

University students often experience numerous financial, social and emotional stressors that can affect their mental health. The Peer Support Centre (PSC) is a pilot project that was established to provide peer support to students in these stressful conditions. We wanted to investigate whether peer support is a viable form of support that would benefit university students. The objective of this study is to determine whether the organization was indeed providing a beneficial service to students and if it was fulfilling the needs of the students that visited the service.

**Methods:**

After a support session, students and peer support providers completed an anonymous questionnaire regarding their self-reported mental wellbeing using the Patient Health Questionnaire-9 (PHQ-9), the Generalized Anxiety Disorder-7 (GAD-7) metrics, and Outcome Rating Scale (ORS). They were also asked about their experience with previous professional mental health services as well as their experience at the PSC. With the data collected from 1043 students and 797 volunteers from September 2016–March 2020, a program evaluation was conducted for quality improvement purposes.

**Results:**

The PSC is used by students of different sexes, genders, and ethnicities. Students reported having a low ORS score, moderate anxiety as per the GAD-7 and moderate depression according to the PHQ-9. They find it easy to use and rely on it as an alternative form of support when they approach barriers that prevent them from accessing professional services. Lastly, the peer support providers feel very validated in their role and overall quite prepared and helpful when helping their fellow peers.

**Conclusions:**

The establishment of a student service that provides peer support would be beneficial to the members of a university/college campus.

**Supplementary Information:**

The online version contains supplementary material available at 10.1186/s13033-021-00479-7.

## Background

University life can be the source of a host of academic, social, financial and cultural challenges for many students [1–3]. These stressors can lead to worsened academic difficulties and/or mental health issues (e.g. anxiety, depression, poor sleep, eating disorders, substance misuse and abuse and/or suicide), ultimately leading to an overall decrease in one’s quality of life [4–10]. Notably, 37–84% of university students who screened positive for depression or anxiety at a university in the US did not consult professional mental health services to address their mental health struggles [11–13]. Moreover, individuals from ages 15 to 24 in Canada are the least likely age group to seek aid for their mental health in the form of professional services, despite being the most affected by mental illness [14].

The reluctance to seek professional services can be attributed to the stigma surrounding mental health issues, low perceived need for help, lack of time, lengthy wait times or waitlists, privacy concerns, or the hierarchical and illness-based approach conducted by clinicians [6, 12–16]. These barriers can lead university students to rely on informal forms of support such as friends and family to help them cope with psychological distress [17]. Knowing why people with mental health struggles do not seek help can aid the development of more effective support systems in university settings to help those students in need. For example, services that provide peer support can serve as an alternative source of informal support for university students due to it being free of cost and conveniently situated on-campus [17, 18].

Peer support is defined as the social and emotional support offered by an individual in equal standing, founded on respect, shared responsibility and mutual agreement of what is helpful [19]. The authenticity found in peer-support relationships can lead to greater feelings of empathy and connectedness as compared to a patient-therapist relationship [20, 21]. Currently available literature shows how participation in peer support workshops and courses leads to improvements in self-esteem, self-acceptance and overall mental wellbeing amongst university students [22, 23]. However, the literature on the benefits of a service that delivers peer support on university campuses is limited due to it being a relatively new phenomenon. Therefore, we investigated whether the peer support service at McGill University was able to meet the needs of the students who used it.

## Methods

### Peer support service

The Peer Support Centre (PSC) is an on-campus, student-led service established at McGill University in downtown Montreal, Quebec. The PSC works closely with the university’s mental health services and professionals to provide free, one-on-one, non-judgemental and non-directional active-listening support to McGill University’s student body. PSC consists of over 100 peer support volunteers (i.e. peer support providers) that undergo rigorous training and assessments to be able to support students during the academic year from September to April. The training program is developed by students based on the community’s needs, and focuses on active listening, open communication, empathy, and crisis management (during individuals’ disclosures of imminent harm to themselves, others or ongoing child abuse). Confidentiality is an essential mandate of the PSC, with the peer support providers and students being required to sign confidentiality agreements prior to initiating a support session. A support session is a safe space in which the student can talk about anything that is on their mind to a peer support provider who will actively listen in an empathetic, non-judgemental and non-directional manner.

### Participants and procedures

Program evaluations are within the mandate of PSC, with no addition of questions or interventions outside the scope of the organization, thus ethical approval from the university’s Research Ethics Board (REB) was not required (TCPS, Article 2.3 and 2.5). Furthermore, the data collected is completely anonymous and the process of data linkage does not generate any identifiable information, thus any secondary use of data that occurs in this study is permitted without approval from the REB (TCPS, Article 2.4). Participants were recruited through the PSC from September 2016 to March 2020 and included all who accessed the service without any exclusion criteria. After a support session, students were invited to fill out an anonymous questionnaire via hard copy or a laptop, with their participation being completely voluntary and confidential. Those who consented to fill out the questionnaire were provided a private space and were permitted to not answer any question on the survey that they felt uncomfortable answering. We have elaborated in the limitations that a student may use this service more than once and that each response to the survey will be counted. This allows us to assess each support session independently from any previous sessions the student may have had in the past. Additionally, for some of the tables (e.g. Table 3), the responses consist of a mixture of the same as well as new peer support providers over time, but with the data leaning towards responses by new providers due to a large turnover each year.

### Measure of mental health status

To assess students’ depressive symptoms over the prior two weeks, nine questions from the Patient Health Questionnaire-9 (PHQ-9) were asked [24]. To assess their experience with anxiety symptoms over the last two weeks, seven questions from the Generalized Anxiety Disorder-7 (GAD-7) were asked [25]. Both the PHQ-9 and GAD-7 are considered to be reliable and valid tools for measuring depression and anxiety respectively [26]. Finally, the students completed the Outcome Rating Scale (ORS) to assess their personal, interpersonal, social and general wellbeing [27]. The ORS is found to be a valid and reliable tool to assess therapeutic outcomes [27]. A more extensive methodology for the survey questions can be found in the Additional file 1.

### Session analysis

In order to assess the quality of the peer support sessions, the Session Rating Scale (SRS) was used [28]. Responses to the four questions range from 0 (“Low agreement”) to 10 (“High agreement”). The sum of the scores were then graded according to the following intervals: potential issues present in the relationship with the peer support provider (0–35), and good relationship between the peer support provider and student (36 +). The SRS is shown to be adequately reliable and valid as a clinical tool [28].

### Qualitative assessment of PSC

Students were asked open-ended questions to find out how they would rate the service that they received at PSC compared to other mental health services that they may have used in the past. Responses were given as a rating from 1 (Terrible)—5 (Excellent) or “Strongly Disagree”—“Strongly Agree”. To assess volunteers’ wellbeing after a support sessions and their feelings of preparedness and helpfulness, they were asked to rank their agreement with shown prompts from 1 (Not at all) to 10 (Yes, very) or 1 (Not at all) to 5 (Yes, a lot). A more extensive methodology for PSC’s qualitative assessment can be found in the Additional file 1.

### Data analysis

The significance of changes between the mean group score of responses over time (weeks, months, and years) was gauged using the Two-Sample t-Test assuming either equal or unequal variances based on the result of a F-test on the sample variances. An alpha value of 0.05 was considered significant. Since all of the sample sizes test were large (at least *N* > 90), we did not test for normality. Data was analyzed using SPSS Statistics, Version 26 (IBM, Armonk, NY) and Microsoft Excel (Microsoft, Redmond, Washington).

## Results

### The PSC serves a wide range of students throughout the school year

**Fig. 1:**
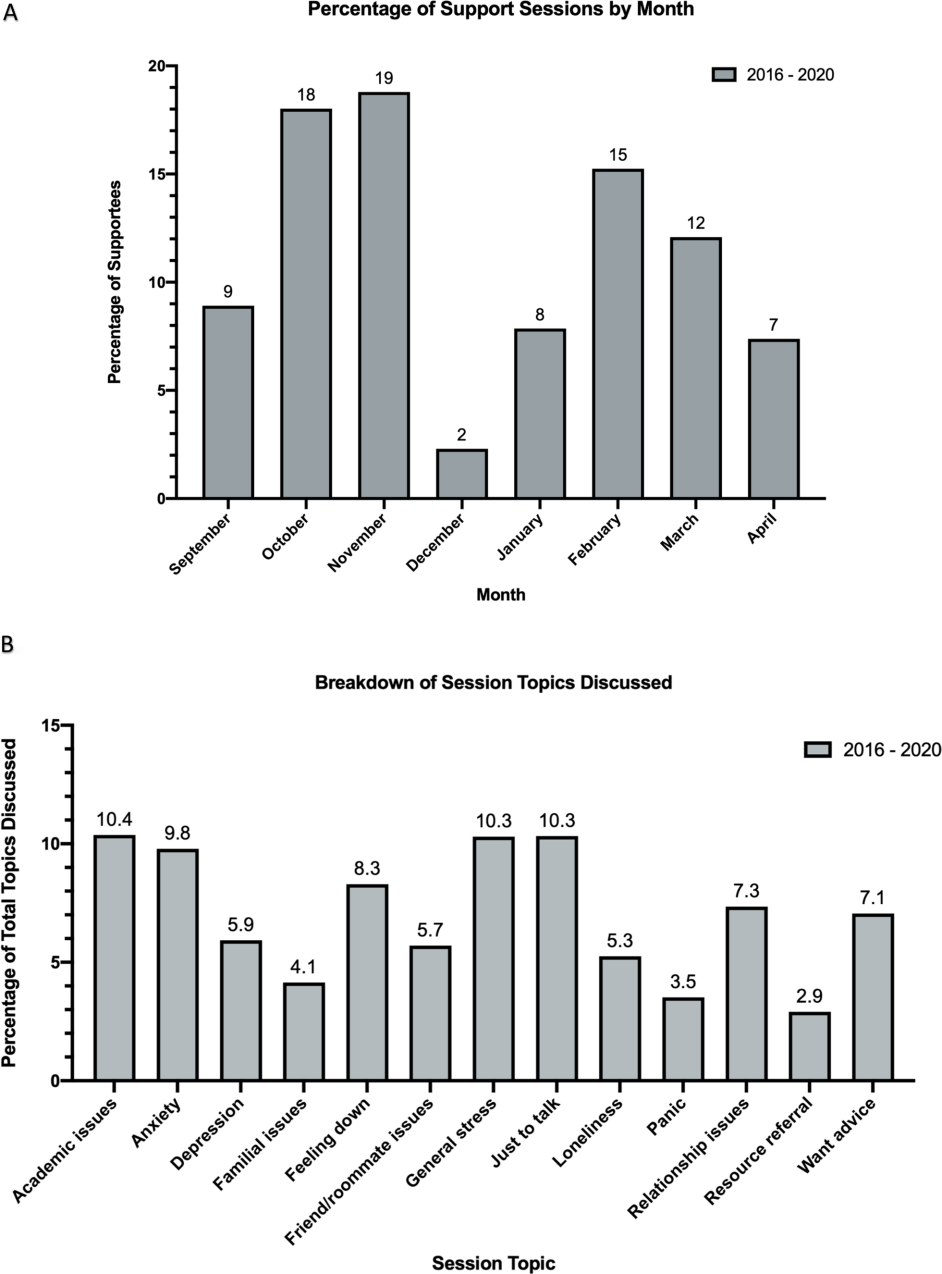
**A** Distribution of the percentage of sessions by month of the academic year from 2016 – 2020. **B** Distribution of topics that came up most (above 2%) in support sessions from 2016 – 2020 (full list can be found in Additional file 5: Table 3)

From September 2016 to March 2020, the PSC provided a total of 1164 support sessions with 950 questionnaires being completed, representing a response rate of 81.6% (Additional file 3: Table S1). During those four academic years, the PSC was visited by students from many different academic departments, ethnic backgrounds, genders, and sexual orientations (Table 1). Students visited this service to a greater extent during the months of October, November, February and March, which coincided with midterm examination periods (Fig. 1A, Additional file 4: Table S2).

**Table 1:** Table showing the demographic breakdown of all the students that used the service from 2016 to 2020

	Total Demographics (2016-2020)
	n (%)
**Gender**	
Male	250 (31.1)
Female	534 (66.5)
Non-binary	14 (1.7)
Prefer not to say	2 (0.2)
Other	4 (0.5)
**Sexual Orientation**	
Homosexual	37 (2.9)
Heterosexual	577 (42.1)
Bisexual	108 (10.4)
Pansexual	29 (1.9)
Asexual	5 (0.3)
Queer	10 (0.8)
Questioning/Unsure	36 (2.9)
Prefer not to answer	2 (0.3)
**Year of Study**	
U0	88 (10.9)
U1	185 (23.0)
U2	168 (20.9)
U3	161 (20.0)
U4	63 (7.8)
Masters	92 (11.4)
PhD	23 (2.9)
Exchange	13 (1.6)
Other	11 (1.4)
**Faculty**	
Agriculture and Environment	36 (4.5)
Arts	337 (41.9)
Arts and Science	25 (3.1)
Dentistry	1 (0.1)
Education	32 (4.0)
Engineering	93 (11.6)
Law	6 (0.7)
Management	59 (7.3)
Medicine	35 (4.3)
Music	21 (2.6)
Science	166 (20.6)
Continuing Studies	1 (0.1)
**Race/ethnicity**	
White/Caucasian/European descent	285 (38.2)
East Asian	187 (25.1)
South Asian	103 (13.8)
West Asian/Middle Eastern	42 (5.6)
Jewish	25 (3.4)
Central/South American	39 (5.2)
African/Black	28 (3.2)
South-East Asian	18 (2.4)
Other/Prefer not to say	6 (0.8)
Indigenous	4 (0.5)
Mixed	13 (1.7)
**In-province, Out-of-province, International Student Status**	
In-province (Quebec)	218 (27.3)
Out-of-province (Canada)	225 (28.3)
International (All)	355 (44.5)
International (US)	114 (14.3)
International (France)	29 (3.6)
International (Other)	212 (26.6)

N = 1043

Notably, as reported by students, they mainly came in for a support session simply to talk to another person, due to academic stress, general stress, feeling anxious, and/or because they were feeling down (Fig. 1B, Additional file 5: Table S3). Interestingly, these session topics were also amongst the top five reported reasons for coming in for a support session during each month of the school year (Additional file 6: Table S4).

### Students’ mental health measures prior to coming to PSC

From September 2018–March 2020, students were asked how they were doing in four different aspects of their lives on the Outcome Rating Scale (ORS). Based on the responses, 234 (77.2%) students had an overall wellbeing score under 25, which indicates that they are experiencing high distress (Additional file 7: Table S5). Interestingly, all of the sections on the ORS during 2019–2020 were lower on average than during 2018–2019 which indicates an overall declining student wellbeing in the rigorous academic environment (P < 0.05, Table 2).

**Table 2:** Table showing the students’ Outcome Rating Scale (ORS), Generalized Anxiety Disorder-7 (GAD-7), Patient Health Questionnaire-9 (PHQ-9), and Session Rating Scale (SRS) measures during each year from 2018 to 2020

	2018–2019			2019–2020		
	Mean (SD)	Mode (n)	Range	Mean (SD)	Mode (n)	Range
**Area** **of** **Wellbeing** **(ORS)**						
Total ORS Score *	21.11 (6.81)	20 (19)	5–40	17.78 (6.54)	23 (9)	4–32
**Area of Anxiety** **(GAD-7)**						
Total anxiety score	10.71 (5.34)	7 (18)	0–21	11.21 (5.40)	7 (11)	0–21
**Area** **of** **Depression** **(PHQ-9)**						
Total depression score	11.51 (6.16)	5 and 13 (15)	0–27	12.50 (6.92)	9 and 10 (9)	0–27
**Aspect of the Session** **(SRS)**						
Relationship—I felt heard, understood, and respected	9.07 (1.58)	10 (126)	1–10	9.20 (1.10)	10 (55)	5–10
Topics—We talked about what I wanted to talk about	9.21 (1.34)	10 (122)	1–10	9.46 (1.08)	10 (71)	5–10
Approach or Method—The peer support provider's approach was a good fit for me	8.56 (1.78)	10 (96)	2–10	8.72 (1.60)	10 (45)	4–10
Relationship—I felt heard, understood, and respected	9.07 (1.58)	10 (126)	1–10	9.20 (1.10)	10 (55)	5–10
Topics—We talked about what I wanted to talk about	9.21 (1.34)	10 (122)	1–10	9.46 (1.08)	10 (71)	5–10

N = 321

*P < 0.05

In order to gauge their levels of anxiety, students were asked questions regarding their level of anxiety over the last 2 weeks. Based on their responses to the GAD-7, 34 (12.9%) of students reported experiencing minimal anxiety, 81 (30.8%) reported mild anxiety, 85 (32.3%) reported moderate anxiety, and 63 (24.0%) reported severe anxiety (Additional file 7: Table S5). None of the areas of anxiety were significantly different from 2018–2019 to 2019–2020 (Table 2).

Next, we sought to gain insight on their levels of depression and feeling low in the two weeks prior to coming to the PSC based on the PHQ-9. Overall, 162 (59.5%) reported experiencing moderate levels of depression or worse (Additional file 7: Table S5). Notably, the proportion of students who felt as though they would be better off dead or hurt in some way was significantly higher during 2019–2020 than 2018–2019 (P < 0.05, Table 2).

### PSC as an alternative source of support for students’ mental health needs

**Fig. 2:**
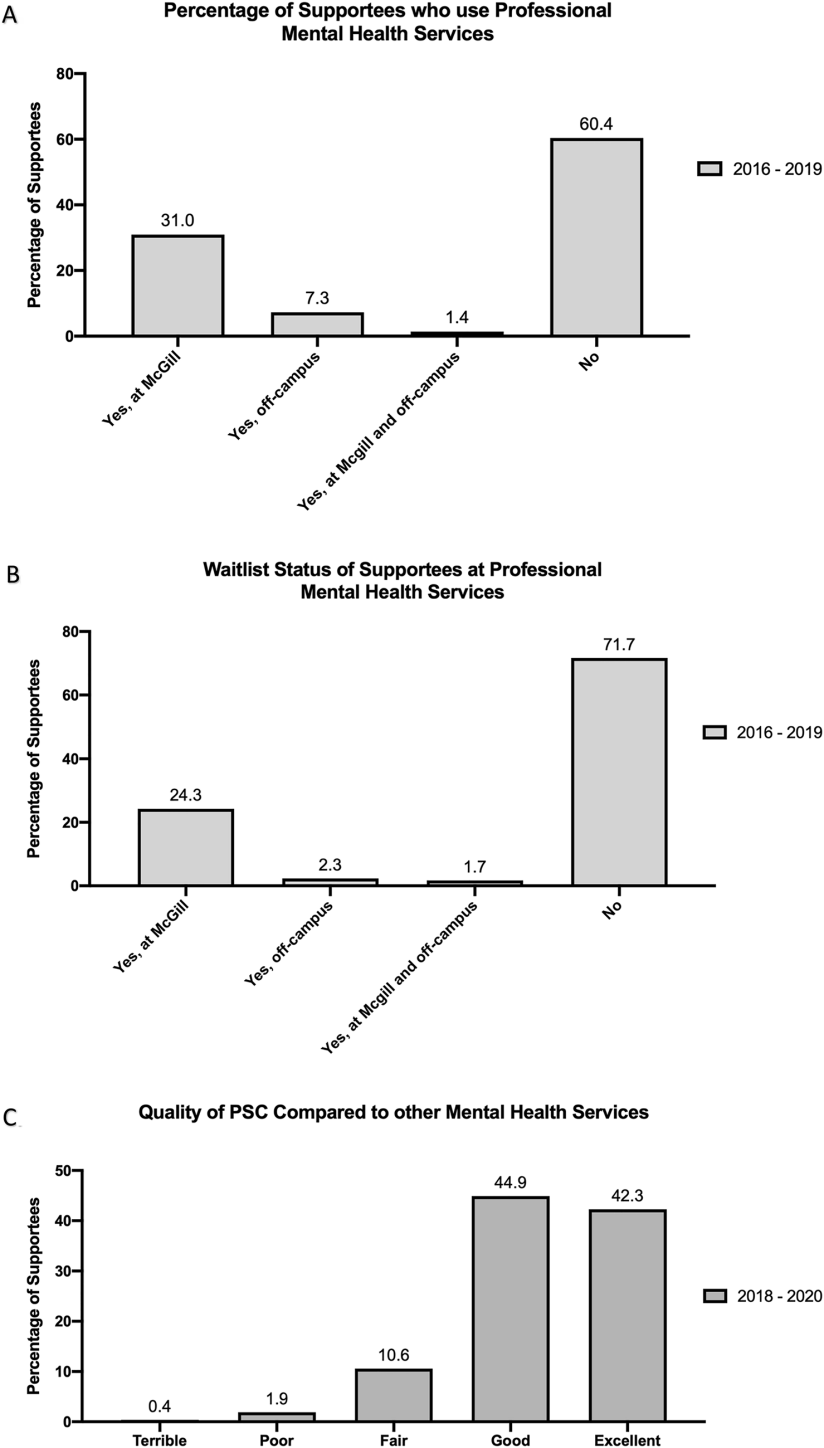
**A** Percentage breakdown of students who use professional mental health services from 2016 to 2019. **B** Percentage breakdown of students who are on a waitlist at professional mental health services from 2016 to 2019. **C** Percentage breakdown of how students would rate the quality of PSC compared to other mental health services

In order to better understand how students addressed their low levels of mental wellbeing, they were asked questions regarding their experience with other mental health services. It was reported from September 2016 to April 2019 that 398 (60.4%) students coming into the PSC did not consult any other professional service (Fig. 2A, Additional file 8: Table S6). Furthermore, 69.7% (n = 182) of those students who were using another professional mental service were on a waitlist either at the McGill campus and/or elsewhere off-campus (Fig. 2B, Additional file 9: Table S7). Additionally, from September 2018 – March 2020, 87.2% (n = 231) of students rated their experience at the PSC from Good to Excellent compared to other mental health services that they were accessing, and only 2.3% (n = 6) of students reported it as being Terrible or Poor. (Fig. 2C, Additional file 10: Table S8).

### Students’ opinions on their visit to the PSC

From September 2018 to March 2020, the mean rating of sessions based on the SRS was found to be 35.9 (S.D. = 5.63), with a mode of 40 (n = 202), which indicates high peer support session quality (Table 2, Additional file 7: Table S5). Additionally, 269 (88.8%) students felt that their visit to the PSC helped them with their emotional and mental wellbeing, with a mean of 4.2 (S.D. = 0.73) and a mode of 4 (n = 158, Additional file 12: Table S10). From 2016 to 2018, 548 (91.6%) students felt that the peer support provider understood what they were experiencing, 456 (76.5%) felt that their peer support provider helped them realize their own resilience and/or coping skills, 420 (70.9%) felt that they were pointed towards other possible resources or services in a helpful way, and 453 (76.1%) felt more equipped to face their circumstances after their support session (Additional file 2: Figs. 1A–D, Additional file 13: Table S11). From 2018 to 2020, 249 (84.9%) students found it relatively simple to navigate the PSC service, 174 (65.7%) didn’t feel that there were many barriers associated with accessing the PSC, and 249 (89%) perceived the PSC as being beneficial to other students on campus (Additional file 2: Figs. 1E–G, Additional file 14: Table S12). Overall, from 2016 to 2020, it turns out that 820 (93.5%) students would recommend the PSC as a service to a friend or a classmate (Additional file 2: Fig. 1H, Additional file 15: Table S13).

### Volunteers’ ability to provide support and their post-session well-being

Data on the peer support providers’ post-session wellbeing was collected from September 2016 – March 2020, with a response rate of 68.5% (n = 797, Additional file 15: Table S13). In terms of preparedness, 698 (88.2%) peer support providers felt quite prepared for the topics that came up during the session, with only 16 (2.0%) peer support providers having felt unprepared (Additional file 16: Table S14). In terms of helpfulness, 540 (73.7%) peer support providers felt that they were very helpful, with only 29 (4.0%) not having felt helpful to the student (Additional file 16: Table S14). There was no statistically significant change in the peer support providers’ feelings of helpfulness from 2018–2019 to 2019–2020 (Additional file 16: Table S14). From September 2018–March 2020, after a session, peer support providers felt very validated in their role (8.22 ± 1.61, mean ± SD on a scale from 1 to 10) and rarely felt conflicted about blurring the line between being a peer support provider and being a potential friend (2.49 ± 2.00), with them being more aware of what their role entails going from 2018–19 to 2019–20 (from 2.70 (2.14) to 2.15 (1.69), mean (SD), P < 0.05, Table 3).

**Table 3:** Table of volunteers’ mental wellbeing scores from 2018 to 2020

Area of Peer support provider’s Wellbeing	2018–2019			2019–2020			Total (2018–2020)		
	Mean (SD)	Mode (n)	Range	Mean (SD)	Mode (n)	Range	Mean (SD)	Mode (n)	Range
I feel validated in my role as a peer support provider	8.22 (1.62)	10 (50)	3–10	8.24 (1.60)	9 (41)	2–10	8.22 (1.61)	10 (85)	2–10
I felt conflicted about how much advice to give	3.99 (2.68)	1 (29)	1–10	3.71 (2.57)	2 (32)	2–10	3.88 (2.64)	1 (75)	1–10
I felt conflicted about being a peer support provider vs. being a potential friend. *	2.70 (2.14)	1 (86)	1–10	2.15 (1.69)	1 (64)	1–9	2.49 (2.00)	1 (150)	1–10
I feel frustrated or sad that I may not see the student again and see how they will be in the future	3.63 (2.49)	1 (55)	1–10	3.74 (2.61)	1 (32)	1–10	3.67 (2.53)	1 (87)	1–10
I felt out of my depth because of the intensity of the students feelings or needs	2.71 (2.14)	1 (87)	1–10	2.47 (1.96)	1 (61)	1–9	2.62 (2.07)	1 (148)	1–10
I am worried about the safety of my student	1.94 (1.57)	1 (125)	1–8	2.15 (1.750	1 (69)	1–10	2.02 (1.64)	1 (194)	1–10

N = 345

*P < 0.05

## Discussion

Through this program evaluation, it is evident that the PSC is accessed by a diverse range of individuals. Notably, more females used the service than males, which can in part be explained by females making up a larger part of the student body than males at McGill [29]. This could also be due to males’ greater perceived self-stigma surrounding help-seeking behaviours, lower mental health literacy, and conflicting ideas about masculinity [30–33]. Additionally, the PSC is visited by a larger proportion of non-White and international students compared to White or Canadian residents, which could be due to them having smaller support networks, experiencing more difficulty adjusting to their new sociocultural environment, and potentially experiencing more discrimination than their Caucasian/local peers [34, 35]. Interestingly, a much larger proportion of bisexual individuals and a lower proportion of heterosexual individuals visited the PSC relative to the McGill population [36], which could possibly be because sexual minority groups tend to face more discrimination and victimization than other groups [37]. These findings suggest that PSC should work to increase service accessibility, as well as continue to enforce and advertise their open, welcoming and non-judgemental mandate to ensure that they are reaching a greater number of students and demographic groups in need.

Months with more academic assessments, assignments and evaluations are not only stressful by themselves, but when compounded with decreased classmate and teacher support, it has been correlated with increased mental health difficulties in students, and thus this could be a potential reason for students seeking support from a peer-based service [38]. In terms of student wellness, each aspect of the ORS measure declined going from 2018–2019 to 2019–2020, and although there wasn’t a drastic change at McGill in terms of how classes were run, studies have shown that depressive symptoms and burnout are increasing amongst college students [39]. This is also consistent with our findings of a larger number of upper-year university students using our service as compared to first-year students. This finding may be preliminary evidence indicating that the mental health of university students is indeed deteriorating over time. Unfortunately, there was an increase in the proportion of students with suicidal thoughts, and this still remains a critical issue in academic environments which needs to be addressed by academic institutions [40].

Consistent with previous reports, most students do not consult any other professional services to address their mental health needs. This could potentially be due to misconceptions regarding the cost and effectiveness of mental health care [41]. A large percentage of students reported not being on a waitlist or not seeing a professional in the first place. Students rate PSC very highly and easy to access with minimal barriers compared to other mental health services that they have used, which corroborates with previous studies emphasizing the benefits of peer support [12–15, 42, 43]. However, it should be noted that peer support serves only to complement the available professional mental health services rather than replacing them entirely.

Lastly, peer support also benefits the peer support providers through improving their self-esteem and giving them feelings of empowerment [44]. Overall, peer support providers felt as though they were fulfilling their role as a peer support provider quite well and felt that they were able to help the student. Interestingly, peer support providers felt more prepared in 2019 – 2020 compared to 2018 – 2019 (P < 0.05, Additional file 17: Table S15), but were more conflicted about being a peer support provider versus a potential friend (Table 3), which may be due to changes over the years in regards to the PSC’s training programs, along with improved supplemental trainings and more frequent practice sessions throughout the year. However, it should be noted that the responses consist of a mixture of the same as well as new peer support providers over time, but with the data leaning towards responses by new providers due to a large turnover each year.

## Limitations

Students consented to fill out the form under the condition that they were freely allowed to not answer questions that they weren’t comfortable answering (e.g. ethnicity, their use of other mental health services, etc.), so some questions have a smaller number of responses compared to others. Furthermore, since data is anonymous, the same student could have completed the questionnaire multiple times and we are unable to monitor this. A student may use this service more than once but their response each visit is logged in as an individual entry and not linked to any previous ones. This allows for the assessment of each support session individually, separate from any previous sessions that the student may have had. Additionally, the responses are indicative of the short-term effects of a support session, and further studies are required to determine its long-term effects on the mental health of university students. Next, different qualitative questions were asked of the students in 2016–2018 as compared to 2018–2020 since the post-session questions were chosen by the Executive Team of that year. However, all the data used in this study was collected over a minimum period of 2 years for a sufficient sample size and response rate. Another limitation would be the lack of qualitative data collected from the students that used this service beyond those included in Additional files 10, 11, 12, 13, 14: Tables 8–12 and Additional file 2: Figure S1. Future studies should include more of these questions in the survey beyond just the quantitative measures. Lastly, It should be noted that from 2019 – 2020, the dataset only spans till mid-March due to the abrupt campus shutdown as a result of the COVID-19 pandemic which ceased PSC’s services for that academic year.

## Conclusion

Peer support and the establishment of on-campus peer support services can be very beneficial to university and college students in helping them better cope with the numerous stressors in their academic environment. The Peer Support Centre appears to fulfil its mandate of providing empathetic, confidential, non-judgemental and non-directional support to students at McGill University in an accessible manner. Taken together, the establishment of an on-campus peer support service is beneficial and relied upon by students at a university campus.

## Supplementary Information

**Additional file 1**.

**Additional file 2**: **Figure S1.** A-D) Percentage distribution of students’ answers to qualitative prompts asking about their overall experience with PSC, during each year from 2016 – 2018. E-G) Percentage distribution of students’ answers to qualitative prompts asking about their ease of access obtaining a support session and whether they perceive this service as being beneficial to students, during each year from 2018 – 2020. H) Percentage distribution of students’ answers to the qualitative prompts asking whether students would recommend this service to a friend or classmate, during each year from 2016 – 2020.

**Additional file 3**: **Table S1.** Table with the number of questionnaire responses of students by year.

**Additional file 4**: **Table S2.** Table with the number of sessions per month of the academic year.

**Additional file 5**: **Table S3.** Table with number of support sessions that each topic came up during each year from 2016 – 2020.

**Additional file 6**: **Table S4.** Table with the most discussed topics in a support session during each month of the academic year.

**Additional file 7**: **Table S5.** Table showing the students’ Outcome Rating Scale (ORS), Generalized Anxiety Disorder-7 (GAD-7), Patient Health Questionnaire-9 (PHQ-9) and Session Rating Scale (SRS) average measures from 2018 – 2020. N = 321.

**Additional file 8**: **Table S6.** Table with the number of responses to the prompt asking whether students use another professional mental health service, during each year from 2016 – 2019.

**Additional file 9**: **Table S7** Table with the number of responses to the prompt asking whether students on a waitlist to use a professional mental health service, during each year from 2016 – 2019.

**Additional file 10**: **Table S8.** Table with the number of responses to the prompt asking how students would compare the quality of the service that they received at PSC to other mental health services, during each year from 2018 – 2020.

**Additional file 11**: **Table S9.** Table with the number of responses to the prompt asking how students would compare the quality of the service that they received at PSC to other mental health services, during each year from 2018 – 2020.

**Additional file 12**: **Table S10.** Table with the number of responses to the prompts asking about their overall experience with PSC, during each year from 2016 – 2018.

**Additional file 13**: **Table S11.** Table with the number of responses to the prompts asking about their ease of access obtaining a support session and whether they perceive this service as being beneficial to students, during each year from 2018 – 2020.

**Additional file 14**: **Table S12.** Table with the number of responses to the prompt asking whether students would recommend this service to a friend or classmate, during each year from 2016 – 2020.

**Additional file 15**: **Table S13**. Table with the number of questionnaire responses of volunteers (peer support providers) by year.

**Additional file 16**: **Table S14.** Table with the number of responses to the prompt asking prepared or helpful volunteers felt when conducting a support session, during each year from 2016 – 2020.

**Additional file 17**: **Table S15**: Table with the means, modes and ranges of peer support providers’ preparedness and helpfulness rating during each year from 2018 – 2020. N = 797, *P < 0.05.

## Data Availability

All data generated or analysed during this study are included in this published article [and its Additional files].
