# Supplementary material for: Program evaluation of a student-led peer support service at a Canadian university
Source: Int J Ment Health Syst. 2021 May 31;15:54. doi: 10.1186/s13033-021-00479-7 (PMC8165510; doi:10.1186/s13033-021-00479-7)
Supplement: Supplementary file 1 — Additional file 1. [file 13033_2021_479_MOESM1_ESM.docx]

**SUPPLEMENTARY METHODS**

***Measure of Mental Health Status***

For both the PHQ-9 and GAD-7, the options for the responses to both sets of questions range from 0 (“Not at all”) to 3 (“Nearly every day”). The sum of the scores for the PHQ-9 was graded based on which interval it fell into: none/minimal (0-5) to severe (20+). The sum of the scores for the GAD-7 questionnaire was graded according to the following range: minimal (0-5) to severe (15+). A question was also asked to assess how difficult these problems made it for the students to get along with other people, to perform academically, and take care of things at home. The responses to these questions range from 0 (“Not difficult at all”) to 3 (“Extremely difficult”). For the ORS, the responses to the questions range from 0 (“Low wellbeing”) to 10 (“High wellbeing”) for each category. The sum of the responses was assessed according to the following intervals: overall low wellbeing (0-24) and average to high wellbeing (25+).

***Qualitative Assessment of PSC***

To assess how students compared the service they received at PSC compared to other mental health services they visited in the past, students were asked the following questions: “Do you use any professional services for your mental health needs?” and “If yes, what is your waitlist status at this service?”. The possible responses were: “Yes, at McGill”, “Yes, off-campus”, “Yes, at McGill and off-campus” and “No”. Additionally, they were asked “How would you compare the quality of PSC compared to other mental health services?” to which they could give a rating from 1 (Terrible) – 5 (Excellent).

To assess students’ experience using the PSC, they were asked to respond to the following prompts: “I felt that my peer support provider understood what I was experiencing”, “I felt that my peer support provider helped me realize my own resilience and/or coping skills”, “I felt that I was pointed towards other possible resources or services in a helpful way”, “I felt more equipped to face my circumstances”, “It was relatively simple to navigate the PSC service”, “There were several barriers associated with accessing the PSC”, “When I first learned about the PSC, I perceived this service as being beneficial for students”, and “I would recommend PSC to a friend or classmate". Students’ responses to these prompts could range from “Strongly Disagree” – “Strongly Agree”.

To assess volunteers’ wellbeing after a support sessions, they were asked to rank their agreement with the following prompts from 1 (Not at all) to 10 (Yes, very): “I feel validated in my role as a peer support provider”, “I felt conflicted about how much advice to give”, “I felt conflicted about being a peer support provider vs. being a potential friend”, “I feel frustrated or sad that I may not see the student again and see how they will be in the future”, “I felt out of my depth because of the intensity of the students feelings or needs”, and “I am worried about the safety of my student”. For their feelings of preparedness and helpfulness, they were asked “Did you feel well-prepared for the topics that came up during the session?” and “Did you feel you were able to help the peer support provider?” Their responses could range from 1 (Not at all) to 5 (Yes, a lot).
