# Supplementary figures and images for: Program evaluation of a student-led peer support service at a Canadian university
Source: Int J Ment Health Syst. 2021 May 31;15:54. doi: 10.1186/s13033-021-00479-7 (PMC8165510; doi:10.1186/s13033-021-00479-7)

A.

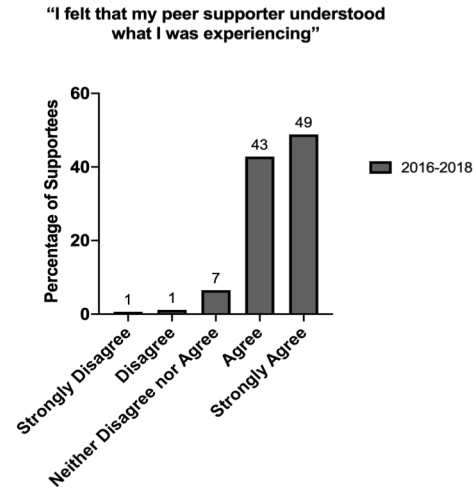

B.

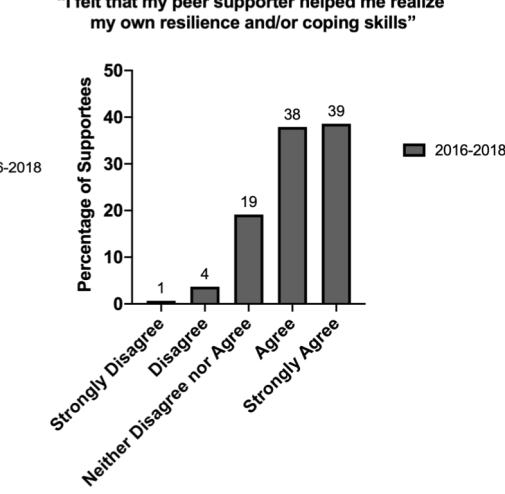

C.

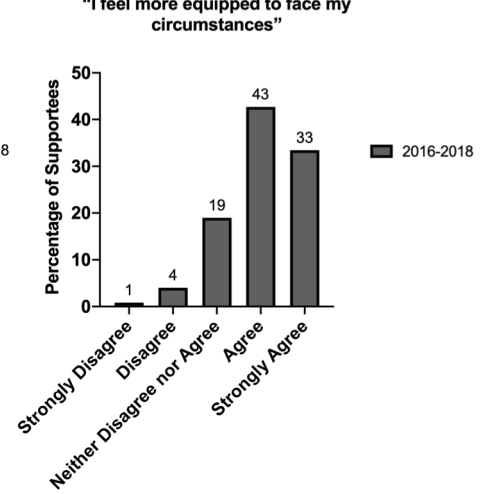

D.

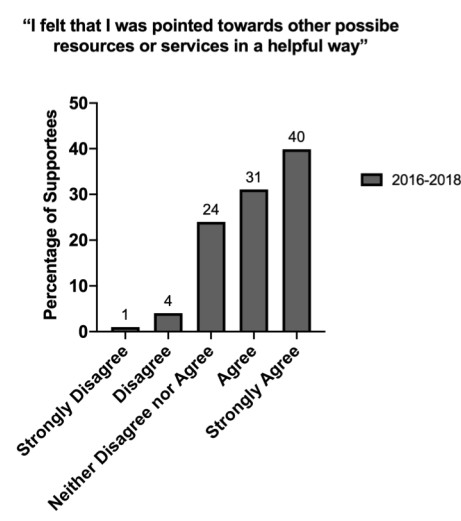

E.

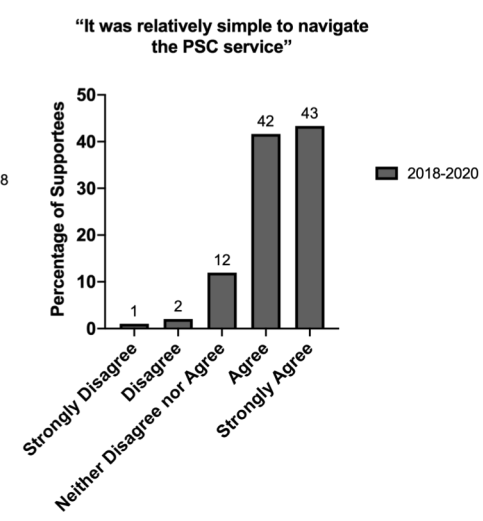

F.

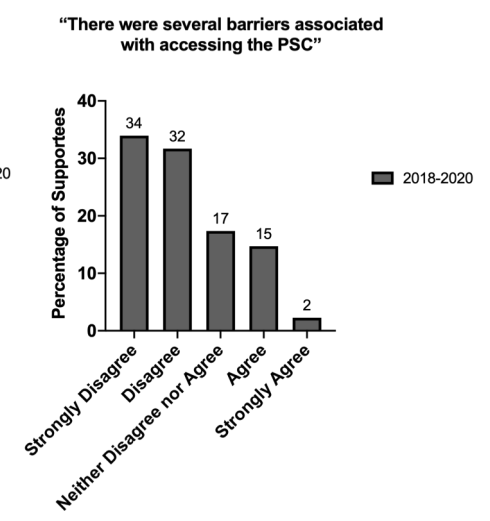

G.

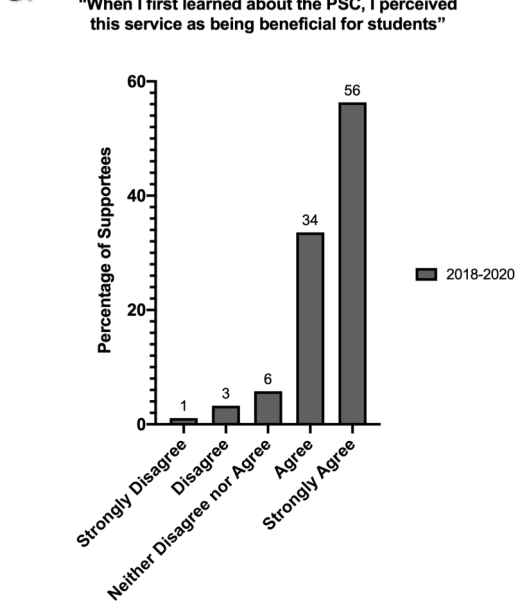

H.

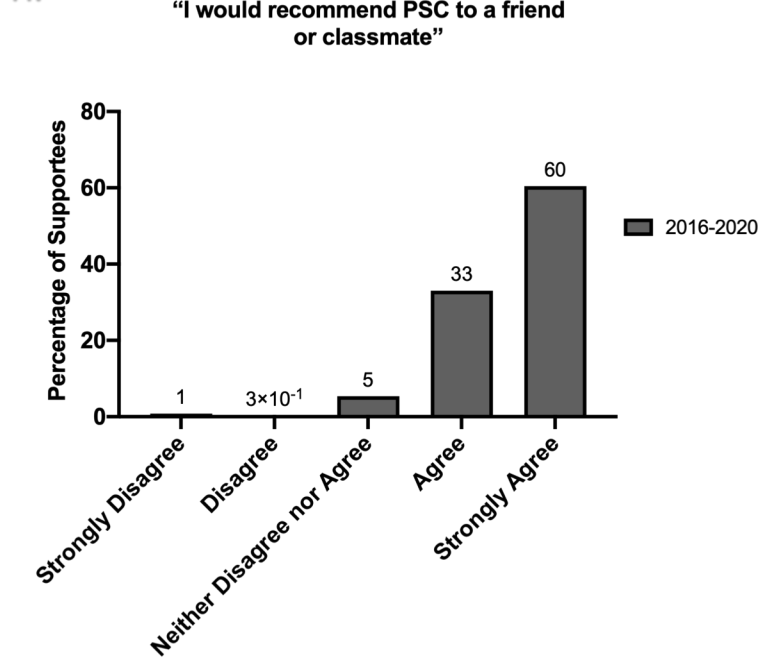

Supplement: Supplementary file 2 — Additional file 2: Figure S1. A-D) Percentage distribution of students’ answers to qualitative prompts asking about their overall experience with PSC, during each year from 2016 – 2018. E-G) Percentage distribution of students’ answers to qualitative prompts asking about their ease of access obtaining a support session and whether they perceive this service as being beneficial to students, during each year from 2018 – 2020. H) Percentage distribution of students’ answers to the qualitative prompts asking whether students would recommend this service to a friend or classmate, during each year from 2016 – 2020. [file 13033_2021_479_MOESM2_ESM.pdf]
