# Supplementary material for: Program evaluation of a student-led peer support service at a Canadian university
Source: Int J Ment Health Syst. 2021 May 31;15:54. doi: 10.1186/s13033-021-00479-7 (PMC8165510; doi:10.1186/s13033-021-00479-7)
Supplement: Supplementary file 3 — Additional file 3: Table S1. Table with the number of questionnaire responses of students by year. [file 13033_2021_479_MOESM3_ESM.docx]

| **Year** | **Number of Sessions** | **Number of Questionnaires Completed** | **Response Rate (%)** |
| --- | --- | --- | --- |
| 2016 – 2017 | 365 | 338 | 92.6 |
| 2017 – 2018 | 348 | 291 | 83.6 |
| 2018 – 2019 | 293 | 223 | 76.1 |
| 2019 – 2020 | 158 | 98 | 62.0 |
| Total (2018 – 2020) | 451 | 321 | 71.2 |
| Total (2016 – 2020) | 1164 | 1043 | 89.6 |
