# Supplementary material for: Program evaluation of a student-led peer support service at a Canadian university
Source: Int J Ment Health Syst. 2021 May 31;15:54. doi: 10.1186/s13033-021-00479-7 (PMC8165510; doi:10.1186/s13033-021-00479-7)
Supplement: Supplementary file 4 — Additional file 4: Table S2. Table with the number of sessions per month of the academic year. [file 13033_2021_479_MOESM4_ESM.docx]

| **Month of the Academic Year** | **Number of Responses** | | | | |
| --- | --- | --- | --- | --- | --- |
|  | **2016 – 2017** | **2017 – 2018** | **2018 – 2019** | **2019 – 2020** | **Total**  **(2016 – 2020)** |
| September | 8 | 41 | 27 | 17 | 93 |
| October | 59 | 53 | 50 | 26 | 188 |
| November | 77 | 50 | 35 | 34 | 196 |
| December | 11 | 9 | 2 | 2 | 24 |
| January | 21 | 36 | 22 | 3 | 82 |
| February | 67 | 51 | 28 | 13 | 159 |
| March | 67 | 24 | 32 | 3 | 126 |
| April | 28 | 27 | 22 | 0 | 77 |
