# Supplementary material for: Program evaluation of a student-led peer support service at a Canadian university
Source: Int J Ment Health Syst. 2021 May 31;15:54. doi: 10.1186/s13033-021-00479-7 (PMC8165510; doi:10.1186/s13033-021-00479-7)
Supplement: Supplementary file 5 — Additional file 5: Table S3. Table with number of support sessions that each topic came up during each year from 2016 – 2020. [file 13033_2021_479_MOESM5_ESM.docx]

| **Topic** | **Number of Responses** | | | | |
| --- | --- | --- | --- | --- | --- |
|  | **2016 – 2017** | **2017 – 2018** | **2018 – 2019** | **2019 – 2020** | **Total**  **(2016 – 2020)** |
| Just to talk | 150 | 103 | 122 | 83 | 458 |
| Want advice | 146 | 63 | 75 | 29 | 313 |
| Resource referral (Resource advice) | 67 | 24 | 22 | 16 | 129 |
| Academic issues (Academic issues) | 215 | 128 | 61 | 56 | 460 |
| General stress | 212 | 133 | 65 | 47 | 457 |
| Familial issues (Family) | 69 | 46 | 31 | 38 | 184 |
| Relationship issues (Relationships) | 137 | 98 | 51 | 40 | 326 |
| Loneliness | 44 | 67 | 69 | 53 | 233 |
| Depression (Depression/severe down mood) | 96 | 65 | 72 | 30 | 263 |
| Suicidality (Suicidal ideation) | 20 | 10 | 4 | 12 | 46 |
| Self-harm | 11 | 0 | 1 | 4 | 16 |
| Grief | 24 | 23 | 8 | 7 | 62 |
| Anxiety | 177 | 126 | 85 | 46 | 434 |
| Panic | 75 | 43 | 28 | 10 | 156 |
| Health issues | 14 | 6 | 9 | 14 | 43 |
| Eating problems (Eating troubles) | 21 | 12 | 3 | 3 | 39 |
| Sexual assault | 0 | 3 | 6 | 8 | 17 |
| Substance use/abuse including alcohol | 19 | 4 | 7 | 7 | 37 |
| Culture shock/International student distress |  |  | 11 | 19 | 30 |
| Sex/gender issues (Gender identity) | 1 | 0 | 4 | 15 | 20 |
| Gambling | 0 | 0 | 0 | 0 | 0 |
| Procrastination |  |  | 25 | 7 | 32 |
| Friend/roommate issues (Friendships/social life) | 137 | 96 | 0 | 20 | 253 |
| Mental difficulties | N/A | N/A | 42 | N/A | 42 |
| Internet issues | N/A | N/A | 1 | N/A | 1 |
| Feeling down | 252 | 116 | N/A | N/A | 368 |
| Suicidal attempt or plan | 6 | N/A | N/A | N/A | 6 |
| Self-harm ideation | 11 | N/A | N/A | N/A | 11 |
