# Supplementary material for: Program evaluation of a student-led peer support service at a Canadian university
Source: Int J Ment Health Syst. 2021 May 31;15:54. doi: 10.1186/s13033-021-00479-7 (PMC8165510; doi:10.1186/s13033-021-00479-7)
Supplement: Supplementary file 6 — Additional file 6: Table S4. Table with the most discussed topics in a support session during each month of the academic year. [file 13033_2021_479_MOESM6_ESM.docx]

| **Month of the Academic Year** | **Most Common Session Topics (% of the month’s topics)** |
| --- | --- |
| September | 1. Just to talk (11.3%) 2. Anxiety (10.3%) 3. General stress (10.1%) 4. Academic issues (8.9%) 5. Want advice (7.6%) |
| October | 1. Anxiety (11.0%) 2. Just to talk (10.2%) 3. General stress (9.8%) 4. Academic issues (9.6%) 5. Feeling down (7.1%) |
| November | 1. General stress (11.2%) 2. Academic issues (10.9%) 3. Just to talk (10.4%) 4. Anxiety (8.7%) 5. Feeling down (8.4%) |
| December | 1. Academic Issues (12.3%) 2. General stress (12.3%) 3. Feeling down (12.3%) 4. Want advice (10.7%) 5. Anxiety (9.8%) |
| January | 1. Academic issues (9.8%) 2. Want advice (10.7%) 3. Anxiety (9.8%) 4. Just to talk (9.0%) 5. General stress (8.9%) |
| February | 1. General stress (11.9%) 2. Feeling down (11.0%) 3. Just to talk (9.7%) 4. Academic issues (9.7%) 5. Anxiety (9.6%) |
| March | 1. Academic issues (11.7%) 2. Just to talk (11.4%) 3. Anxiety (10.0%) 4. Feeling down (9.0%) 5. General stress (8.9%) |
| April | 1. Academic issues (11.8%) 2. Just to talk (10.3%) 3. General stress (9.7%) 4. Anxiety (9.4%) 5. Feeling down (8.5%) |
