# Supplementary material for: Program evaluation of a student-led peer support service at a Canadian university
Source: Int J Ment Health Syst. 2021 May 31;15:54. doi: 10.1186/s13033-021-00479-7 (PMC8165510; doi:10.1186/s13033-021-00479-7)
Supplement: Supplementary file 7 — Additional file 7: Table S5. Table showing the students’ Outcome Rating Scale (ORS), Generalized Anxiety Disorder-7 (GAD-7), Patient Health Questionnaire-9 (PHQ-9) and Session Rating Scale (SRS) average measures from 2018 – 2020. N = 321. [file 13033_2021_479_MOESM7_ESM.docx]

| **ORS** | **Area of Wellbeing** | **2018 - 2020** | | |
| --- | --- | --- | --- | --- |
|  |  | **Mean (SD)** | **Mode (n)** | **Range** |
|  | Individually | 4.94 (1.97) | 5 (63) | 1-10 |
|  | Interpersonally | 5.18 (2.08) | 5 (64) | 1-10 |
|  | Socially | 4.92 (2.07) | 5 and 6 (55) | 1-10 |
|  | Overall | 4.95 (1.84) | 5 (84) | 1-10 |
|  | Total ORS Score | 19.98 (6.89) | 20 (26) | 4-40 |
|  |  |  |  |  |
| **GAD-7** | **Area of Anxiety** | **2018 - 2020** | | |
|  |  | **Mean (SD)** | **Mode (n)** | **Range** |
|  | Nervousness | 1.86 (0.92) | 1 (89) | 0-3 |
|  | Inability to stop worrying | 1.82 (0.97) | 1 (90) | 0-3 |
|  | Worrying too much | 1.91 (0.89) | 1 (86) | 0-3 |
|  | Trouble relaxing | 1.75 (0.96) | 1 (97) | 0-3 |
|  | Being so restless it is hard to stand still | 0.94 (1.01) | 0 (115) | 0-3 |
|  | Being easily annoyed or irritable | 1.18 (1.00) | 1 (104) | 0-3 |
|  | Feeling afraid something awful might happen | 1.38 (1.09) | 1 (88) | 0-3 |
|  | Total anxiety score | 10.83 (5.33) | 7 (29) | 0-21 |
|  | Impairment severity | 1.61 (0.81) | 1 (111) | 0-3 |
|  |  |  |  |  |
| **PHQ-9** | **Area of Depression** | **2018 - 2020** | | |
|  |  | **Mean (SD)** | **Mode (n)** | **Range** |
|  | Little interest or pleasure in doing things | 1.28 (0.93) | 1 (122) | 0-3 |
|  | Feeling down, depressed, or hopeless | 1.53 (0.94) | 1 (118) | 0-3 |
|  | Trouble falling/staying asleep, sleeping too much | 1.54 (1.02) | 1 (102) | 0-3 |
|  | Feeling tired or having little energy | 1.69 (0.93) | 1 (110) | 0-3 |
|  | Poor appetite or overeating | 1.22 (1.04) | 1 (99) | 0-3 |
|  | Feeling bad about yourself, or that you're a failure, or have let yourself or your family down | 1.63 (1.00) | 1 (104) | 0-3 |
|  | Trouble concentrating on things such as reading | 1.47 (1.00) | 1 (101) | 0-3 |
|  | Being so fidgety or restless that you have been moving around more than usual | 0.98 (1.06) | 0 (122) | 0-3 |
|  | Thoughts that you would be better off dead or of hurting yourself in some way | 0.50 (0.83) | 0 (181) | 0-3 |
|  | Total depression score | 11.74 (6.40) | 10 (23) | 0-27 |
|  | Impairment severity | 1.61 (0.84) | 1 (116) | 0-3 |
|  |  |  |  |  |
| **SRS** | **Aspect of the Session** | **2018 - 2020** | | |
|  |  | **Mean (SD)** | **Mode (n)** | **Range** |
|  | Relationship - I felt heard, understood, and respected. | 9.14 (1.43) | 10 (181) | 1-10 |
|  | Topics - We talked about what I wanted to talk about. | 9.30 (1.26) | 10 (193) | 1-10 |
|  | Approach or Method - The peer-peer support provider's approach was a good fit for me. | 8.69 (1.72) | 10 (141) | 2-10 |
|  | Overall - Overall, today's session was right for me. | 8.79 (1.78) | 10 (152) | 2-10 |
|  | Total | 35.88 (5.41) | 40 (239) | 6-40 |
