# Supplementary material for: Program evaluation of a student-led peer support service at a Canadian university
Source: Int J Ment Health Syst. 2021 May 31;15:54. doi: 10.1186/s13033-021-00479-7 (PMC8165510; doi:10.1186/s13033-021-00479-7)
Supplement: Supplementary file 8 — Additional file 8: Table S6. Table with the number of responses to the prompt asking whether students use another professional mental health service, during each year from 2016 – 2019. [file 13033_2021_479_MOESM8_ESM.docx]

| **Students who use a professional mental health service** | **Number of Responses** | | | |
| --- | --- | --- | --- | --- |
|  | **2016 – 2017** | **2017 – 2018** | **2018 – 2019** | **Total (2016 – 2019)** |
| Yes, at McGill | 53 | 86 | 65 | 204 |
| Yes, off-campus | 3 | 23 | 22 | 48 |
| Yes, at McGill and off-campus | 9 | 0 | 0 | 9 |
| No | 103 | 175 | 120 | 398 |
