# Supplementary material for: Program evaluation of a student-led peer support service at a Canadian university
Source: Int J Ment Health Syst. 2021 May 31;15:54. doi: 10.1186/s13033-021-00479-7 (PMC8165510; doi:10.1186/s13033-021-00479-7)
Supplement: Supplementary file 9 — Additional file 9: Table S7 Table with the number of responses to the prompt asking whether students on a waitlist to use a professional mental health service, during each year from 2016 – 2019. [file 13033_2021_479_MOESM9_ESM.docx]

| **Waitlist at a professional mental health service** | **Number of Responses** | | | |
| --- | --- | --- | --- | --- |
|  | **2016 – 2017** | **2017 – 2018** | **2018 – 2019** | **Total (2016 – 2019)** |
| Yes, at McGill | 54 | 80 | 22 | 156 |
| Yes, off-campus | 2 | 8 | 5 | 15 |
| Yes, at McGill and off-campus | 5 | 3 | 3 | 11 |
| No | 108 | 196 | 157 | 461 |
