# Supplementary material for: Program evaluation of a student-led peer support service at a Canadian university
Source: Int J Ment Health Syst. 2021 May 31;15:54. doi: 10.1186/s13033-021-00479-7 (PMC8165510; doi:10.1186/s13033-021-00479-7)
Supplement: Supplementary file 10 — Additional file 10: Table S8. Table with the number of responses to the prompt asking how students would compare the quality of the service that they received at PSC to other mental health services, during each year from 2018 – 2020. [file 13033_2021_479_MOESM10_ESM.docx]

| **Quality compared to other services** | **Number of Responses** | | |
| --- | --- | --- | --- |
|  | **2018 – 2019** | **2019 – 2020** | **Total (2018 – 2020)** |
| 1 (Terrible) | 0 | 1 | 1 |
| 2 (Poor) | 2 | 3 | 5 |
| 3 (Fair) | 16 | 12 | 28 |
| 4 (Good) | 91 | 28 | 119 |
| 5 (Excellent) | 77 | 35 | 112 |
