# Supplementary material for: Program evaluation of a student-led peer support service at a Canadian university
Source: Int J Ment Health Syst. 2021 May 31;15:54. doi: 10.1186/s13033-021-00479-7 (PMC8165510; doi:10.1186/s13033-021-00479-7)
Supplement: Supplementary file 11 — Additional file 11: Table S9. Table with the number of responses to the prompt asking how students would compare the quality of the service that they received at PSC to other mental health services, during each year from 2018 – 2020. [file 13033_2021_479_MOESM11_ESM.docx]

| **Year** | **Mean (SD)** | **Mode (n)** | **Range** |
| --- | --- | --- | --- |
| 2018 – 2019 | 4.27 (0.69) | 4 (111) | 1-5 |
| 2019 – 2020 | 4.14 (0.79) | 4 (47) | 1-5 |
| Total (2018 – 2020) | 4.23 (0.73) | 4 (158) | 1-5 |
