# Supplementary material for: Program evaluation of a student-led peer support service at a Canadian university
Source: Int J Ment Health Syst. 2021 May 31;15:54. doi: 10.1186/s13033-021-00479-7 (PMC8165510; doi:10.1186/s13033-021-00479-7)
Supplement: Supplementary file 12 — Additional file 12: Table S10. Table with the number of responses to the prompts asking about their overall experience with PSC, during each year from 2016 – 2018. [file 13033_2021_479_MOESM12_ESM.docx]

| **Prompt + Rating** | **Number of Responses** | | |
| --- | --- | --- | --- |
|  | **2016 – 2017** | **2017 – 2018** | **Total (2016 – 2018)** |
| I felt that my peer support provider understood what I was experiencing.  Strongly Disagree  Disagree  Neither Disagree nor Agree  Agree  Strongly Agree | 3  2  21  131  154 | 1  5  18  125  138 | 4  7  39  256  292 |
| I felt that my peer support provider helped me realize my own resilience and/or coping skills.  Strongly Disagree  Disagree  Neither Disagree nor Agree  Agree  Strongly Agree | 3  8  66  102  131 | 1  14  48  124  99 | 4  22  114  226  230 |
| I felt that I was pointed towards other possible resources or services in a helpful way.  Strongly Disagree  Disagree  Neither Disagree nor Agree  Agree  Strongly Agree | 3  8  61  89  146 | 3  16  81  95  90 | 6  24  142  184  236 |
| I feel more equipped to face my circumstances.  Strongly Disagree  Disagree  Neither Disagree nor Agree  Agree  Strongly Agree | 3  11  63  108  123 | 2  13  50  146  76 | 5  24  113  254  199 |
