# Supplementary material for: Program evaluation of a student-led peer support service at a Canadian university
Source: Int J Ment Health Syst. 2021 May 31;15:54. doi: 10.1186/s13033-021-00479-7 (PMC8165510; doi:10.1186/s13033-021-00479-7)
Supplement: Supplementary file 13 — Additional file 13: Table S11. Table with the number of responses to the prompts asking about their ease of access obtaining a support session and whether they perceive this service as being beneficial to students, during each year from 2018 – 2020. [file 13033_2021_479_MOESM13_ESM.docx]

| **Prompt + Rating** | **Number of Responses** | | |
| --- | --- | --- | --- |
|  | **2018 – 2019** | **2019 – 2020** | **Total**  **(2019 – 2020)** |
| It was relatively simple to navigate the PSC service.  Strongly Disagree  Disagree  Neither Disagree nor Agree  Agree  Strongly Agree | 3  6  29  98  70 | 0  0  6  24  57 | 3  6  35  122  127 |
| There were several barriers associated with accessing the PSC.  Strongly Disagree  Disagree  Neither Disagree nor Agree  Agree  Strongly Agree | 40  71  38  35  3 | 50  13  8  4  3 | 90  84  46  39  6 |
| When I first learned about the PSC, I perceived this service as being beneficial for students.  Strongly Disagree  Disagree  Neither Disagree nor Agree  Agree  Strongly Agree | 2  5  11  70  108 | 1  4  5  23  48 | 3  9  16  93  156 |
