# Supplementary material for: Program evaluation of a student-led peer support service at a Canadian university
Source: Int J Ment Health Syst. 2021 May 31;15:54. doi: 10.1186/s13033-021-00479-7 (PMC8165510; doi:10.1186/s13033-021-00479-7)
Supplement: Supplementary file 14 — Additional file 14: Table S12. Table with the number of responses to the prompt asking whether students would recommend this service to a friend or classmate, during each year from 2016 – 2020. [file 13033_2021_479_MOESM14_ESM.docx]

| **Prompt + Rating** | **Number of Responses** | | | | |
| --- | --- | --- | --- | --- | --- |
|  | **2016 – 2017** | **2017 - 2018** | **2018 – 2019** | **2019 – 2020** | **Total**  **(2016 – 2020)** |
| I would recommend PSC to a friend or classmate.  Strongly Disagree  Disagree  Neither Disagree nor Agree  Agree  Strongly Agree | 2  0  19  91  195 | 1  2  13  109  161 | 3  0  6  70  123 | 1  1  9  20  51 | 7  3  47  290  530 |
