# Supplementary material for: Program evaluation of a student-led peer support service at a Canadian university
Source: Int J Ment Health Syst. 2021 May 31;15:54. doi: 10.1186/s13033-021-00479-7 (PMC8165510; doi:10.1186/s13033-021-00479-7)
Supplement: Supplementary file 16 — Additional file 16: Table S14. Table with the number of responses to the prompt asking prepared or helpful volunteers felt when conducting a support session, during each year from 2016 – 2020. [file 13033_2021_479_MOESM16_ESM.docx]

| **Rating** | **Number of Responses** | | | | |
| --- | --- | --- | --- | --- | --- |
|  | **2016 – 2017** | **2017 – 2018** | **2018 – 2019** | **2019 – 2020** | **Total**  **(2016 – 2020)** |
| Preparedness  1 (Very Unprepared)  2 (Somewhat Unprepared)  3 (Neither Unprepared nor Prepared)  4 (Somewhat Prepared)  5 (Very Prepared) | 0  3  16  79  89 | 2  2  28  122  109 | 0  3  10  49  68 | 5  1  23  112  70 | 7  9  77  362  336 |
| Helpfulness  1 (Not very Helpful)  2 (Somewhat Unhelpful)  3 (Neither Unhelpful nor Helpful)  4 (Somewhat Helpful)  5 (Very Helpful) | 1  5  43  93  45 | 2  11  54  133  6 | 0  6  23  65  36 | 2  2  44  104  58 | 5  24  164  395  145 |
