# Supplementary material for: Program evaluation of a student-led peer support service at a Canadian university
Source: Int J Ment Health Syst. 2021 May 31;15:54. doi: 10.1186/s13033-021-00479-7 (PMC8165510; doi:10.1186/s13033-021-00479-7)
Supplement: Supplementary file 17 — Additional file 17: Table S15: Table with the means, modes and ranges of peer support providers’ preparedness and helpfulness rating during each year from 2018 – 2020. N = 797, *P < 0.05. [file 13033_2021_479_MOESM17_ESM.docx]

| **Category** | **2018 – 2019** | | | **2019 - 2020** | | |
| --- | --- | --- | --- | --- | --- | --- |
|  | **Mean (S.D.)** | **Mode (n)** | **Range** | **Mean (S.D.)** | **Mode (n)** | **Range** |
| **Preparedness*** | 4.14 (0.81) | 4 (112) | 1-5 | 4.40 (0.73) | 5 (68) | 2-5 |
| **Helpfulness** | 4.02 (0.78) | 4 (104) | 1-5 | 4.01 (0.30) | 4 (65) | 2-5 |
